# CD68, CD163, and matrix metalloproteinase 9 (MMP-9) in breast tumor microenvironment to predict breast cancer survival: are they enough?

**DOI:** 10.1186/s13058-019-1134-z

**Published:** 2019-04-03

**Authors:** Sara Bravaccini, Roberta Maltoni

**Affiliations:** Istituto Scientifico Romagnolo per lo Studio e la Cura dei Tumori (IRST) IRCCS, Meldola, Italy

**Keywords:** CD68, CD163, Matrix metalloproteinase 9, Breast cancer, Prognosis

Pelekanou and colleagues recently evaluated tumor-associated macrophage (TAM) biomarkers (CD68, CD163) and MMP-9 expression in breast cancer (BC) and their role in identifying subclasses of patients who can benefit from TAM-targeting therapies [1]. The authors concluded that the association between high co-expression and co-localization of MMP-9/CD163/CD68 and poor survival in ER+ cancers suggests that these patients may be candidates for macrophage-targeted therapies. We found the paper very interesting but would like to make some comments. Using an in situ approach, Pelekanou and colleagues found that CD68 only correlated with poor survival in ER− patients. They used multivariate analysis to evaluate overall survival. However, apart from age, tumor size, and grade, other validated clinical pathological characteristics that can impact prognosis were not included in the multivariate analysis, e.g., the presence and number of involved lymph nodes, Her 2 status, proliferative activity of the primary tumor, and PAM50 subtype classification. In other words, the poorer prognosis seen in ER− patients may have been due to the presence of other unfavorable clinical pathological characteristics that negatively influence prognosis. The authors did not explain the cutoff they used to define PgR positivity, an important aspect given that the prognostic importance of PgR has been demonstrated in some BC patient subsets [2].

Previous studies have reported contradictory results on the prognostic role of TAMs in BC [3, 4]. One explanation could be the lack of standardized methods for TAM detection, possibly due to different staining methods used in terms of antibodies and platforms. It may also indicate different scoring systems (staining intensity, percentage, or combination of both) for TAM phenotypic characterization, which is usually performed by semi-quantitative methods.

Finally, the authors state that tissue microarray (TMA) may induce under- or over-representation of the marker level because of tumor heterogeneity. Another limit of TMA is the very low quantity of microenvironment that is present in specimens. In fact, usually, only the core of the tumor is selected to obtain a TMA, which suggests that Pelekanou may only have considered immune-inflamed tumors (with TAMs inside the tumor tissue), excluding immune-escape tumors whose TAMs are outside the tumor.

We also observed a BC sample with a high number of CD163-positive polarized TAMs outside the tumor using immunohistochemistry (unpublished data).

Another important point that needs to be addressed is whether and how adjuvant therapy impacts conventional clinical parameters and phenotypic macrophage switching (i.e., from M2 to M1). Moreover, in order to plan TAM-targeting treatment, the type of macrophages present in the primary tumor need to be taken into consideration to avoid treating patients who are negative for a specific TAM population with specific TAM-targeting therapies. TAM and other important elements of the tumor microenvironment such as cancer-associated fibroblasts warrant further investigation in prospective studies of patients with homogeneous biological and clinical pathological characteristics to better understand their impact on overall and disease-free survival.
